# Exploring the Barriers and Opportunities for a More Predictive Data-Driven Telecare Service: Qualitative Study in Scotland

**DOI:** 10.2196/85056

**Published:** 2026-02-27

**Authors:** Emma Dunlop, David Kernaghan, Natalie Weir, Kimberley Kavanagh, Marc Roper, Marilyn Lennon

**Affiliations:** 1 Strathclyde Institute of Pharmacy and Biomedical Sciences, University of Strathclyde, 161 Cathedral Street, Glasgow, G4 0RE, United Kingdom; 2 Mathematics and Statistics, University of Strathclyde, 26 Richmond Street, Glasgow, G1 1XH, United Kingdom; 3 Computer and Information Sciences, University of Strathclyde, 26 Richmond Street, Glasgow, G1 1XH, United Kingdom, 44 141 552 4400

**Keywords:** telecare, telehealth, data analytics, predictive models, preventative care

## Abstract

**Background:**

Telecare uses technology to help people live more independently at home. When an adverse event (such as someone falling or a bath overflowing) happens, the technology reactively senses this and alerts a call center to respond. If the technology can detect a person’s current (and past) states and behaviors, with machine learning, we can more proactively identify potential risks before an adverse event occurs and intervene. Despite social care organizations being data-rich, few predictive analytics are currently routinely applied. There is a need to understand current data management practices before optimizing organizational and technical readiness for proactive data-driven telecare services.

**Objective:**

The aim of this study was to understand how specific telecare data (monitoring falls, identifying people at risk of falling, and providing services in response) are collected, managed, and used in the largest health and social care region in Scotland. The objectives were to: (1) map the community alarm data flow to understand what data were being collected, by what services, and where it was stored, linked, and managed; and (2) identify the current barriers and opportunities around staff and organizations using or applying predictive analytics routinely within telecare service provision.

**Methods:**

This qualitative study involved interviews with health and social care professionals working in Glasgow City Council (GCC) and a telecare service provider (Tunstall). Interviews explored experiences of the systems and data access, processes for collecting and using the data, and how it might be better used to target services more proactively. Data underwent a thematic framework analysis. Descriptions of the data flow were used to develop visual representations of the sociotechnical system.

**Results:**

A total of 14 participants at operational and managerial levels took part. A complex sociotechnical telecare system was identified, involving multiple staff roles, with data exchanged across 11 teams, using 17 systems, with 4 distinct data sources. In total, four themes highlighted key challenges that are: (1) suboptimal systems and equipment; (2) data recording inefficiencies and use; (3) specific patient population barriers and IT literacy; and (4) limited resources and support. Opportunities for more predictive telecare included: establishing a more structured and integrated approach to data management; scope for improved data organization and retrieval; better cross-platform integration and data sharing; and the use of tools or models to support insightful data analysis tailored to the users.

**Conclusions:**

Scottish telecare data services require improved infrastructure to be managed in ways that support more predictive telecare services. This includes more structured and linked datasets and greater integration between the services and systems to allow service providers more integrated, up-to-date, and real-time connected data to build accurate and meaningful models.

## Introduction

Technology-enabled care at home services (known as telecare in the United Kingdom) collect behavioral and environmental data from sensors within a person’s home or on the person to: (1) monitor and act to provide a safe environment (eg, community alarms, door alarms to signal wandering risk, and sensors to prevent flooding a bathroom), (2) provide reminders to do something or take action (eg, stand up and take medication), (3) or to look after the home and more [1]. Telecare has long been considered a means of supporting aging populations to maintain independence within their own home [2]. In Scotland, in 2021‐2022, there were an estimated 128,795 people (approximately 1 in 42 people) who used a telecare service or community alarm [3], and 1.8 million people across the United Kingdom accessed telecare [4]. The ability to capture data from devices such as sensors and fall detectors allows for the possibility to monitor both behaviors and outcomes in citizens, and even to predict and prevent negative outcomes, such as falls [5]. This can lead to savings in resources (eg, reduce hospital admissions or ambulance call-outs), reduced carer burden (providing more reliable remote support and reassurance services for family members), and improved citizen well-being and quality of life (allowing people to feel safe, more independent in their own homes [6], and allowing them to live longer in those homes, supported by technology [6,7]).

There is a growing need from service providers and policymakers in health and care globally to use bigger data from more diverse data streams, including from citizens, wearable and mobile technologies, as well as existing routinely collected datasets in health and care [8]. Analysis of these data through the advancement of artificial intelligence (AI) capability could lead to more predictive and proactive models for home care [2] and may have an impact on patient safety and well-being [9]. Within Scotland, for example (the context for this study), the Scottish Government has committed to transitioning the estimated 170,000 citizens in receipt of telecare to a digital service, with a view to improved access to data, future-proofing care, and helping prevent unnecessary and costly hospital admissions [10]. As part of this, telecare manufacturers are working with health and social care partnerships (HSCPs) to collate bigger and better datasets on service users to provide more preventative, rather than reactive, models of care.

Despite the promise AI and machine learning hold within this context, very little predictive analytics have been conducted in the home care setting, as reported by a review by Anderson et al [11]. The review showed much of the research to date has been small-scale and lacking proactive or predictive techniques, instead using simple models of categorizing behaviors, diagnosis of conditions being present or not, or simple anomaly detection (an adverse or undesired event occurring that should be reacted to). To progress the analytic capabilities in this domain of social care and telecare specifically, better understanding is needed of how social care and telecare data are currently being collected and managed, and also to identify how “ready” health and social care organizations are to use these data to build more predictive systems if any of the cost savings and benefits are to be more widely realized.

There are many use cases in telecare and social care more generally, such as predicting the likelihood of, and outcomes from, falls, which was the key use case for this study. Therefore, the aim of this qualitative, explorative study was to understand how data are collected and managed in the largest HSCP in Scotland. The study objectives were to: (1) map the community alarm data flow in that local context to understand the complexity of the different telecare datasets, and how data were transferred, stored, linked, and managed; and (2) identify the current barriers and opportunities around staff and organizations applying predictive analytics in their day-to-day roles within telecare service provision. The research questions are: how are community alarm data collected, managed, and then used within the Glasgow HSCP (data mapping and interviews); and what are the barriers and opportunities around staff and organizations applying predictive analytics within the telecare service in Glasgow HSCP (interviews)?

## Methods

This qualitative study involved interviews with professionals working in the largest HSCP in Scotland (Glasgow City Council [GCC]) and a leading telecare service provider (Tunstall).

### Research Materials

A semistructured interview schedule was developed independently by the research team (Multimedia Appendix 1) and underwent face-validity checking by 2 members of the GCC HSPC team. The schedule included questions covering participants’ experiences of the systems and processes in place for collecting and using telecare data, what data they access, and how data can be better used to help them meet service user and organizational needs. We based our interview schedule on the People, Processes, Technology Framework because we were interested in staff roles and capabilities, the processes and systems for collecting, managing, and using data, and the data and the systems used to manage the data. A participant information sheet (PIS), consent, and demographics forms were developed and hosted online using Qualtrics.

### Recruitment

A purposive sample of eligible participants (n=17) was identified by a member of the project team from GCC HSCP to represent the breadth of staff who might be directly interested in, or working with community and falls alarm data from across their organization and their commercial provider Tunstall. Potential participants included operational staff, including locality or referrer staff, and managerial staff, including senior management. Individuals were contacted by a GCC project team member to invite them to participate. Interested participants were contacted by a researcher via email to arrange an interview. Given the niche nature of the interview criteria seeking people working in specific roles, in a specific organization, stakeholders from GCC provided names and contact details of potential participants. The inclusion criteria were any staff member who was directly involved in, or interested in more predictive analytics usage within the telecare service, or works directly with the data (either collecting it, managing it, or acting on it to improve services). This form of sampling was deemed the most effective method. To counter any risks of bias or influence, GCC were not informed of who accepted or declined participation, nor were they involved in conducting the interviews. Participants were also assured that all comments would be anonymized and that the research team was seeking honest criticism to improve the service. A total of 17 participants were invited by the research team to take part. Of these, 2 did not respond and 1 withdrew with no stated reason, leaving 14 participants to take part in the study.

### Data Collection

Once potential participants had read the online PIS and completed the consent and demographics, one-to-one semistructured interviews were conducted between August and October 2022. Interviews were conducted by a research team independent from GCC and Tunstall. Interviews lasted between 17 and 47 minutes and were conducted remotely using Microsoft Teams, Zoom (Zoom Communications), or telephone. Interviews were recorded via the participant’s preferred platform or with a dictaphone. Participants who could not attend an interview could respond via an online questionnaire using Qualtrics which posed the same questions used during the interview with free-text response options for participants to complete. Participants were offered a copy of the interview schedule before the interview.

### Data Analysis

All interviews were transcribed using an intelligent verbatim approach, using the automatic transcription on the online platform used and edited for accuracy. Free-text data from the interviews and questionnaires were combined into a single dataset and analyzed alongside the interview transcriptions by the research team, independent from the vendors (GCC). GCC did not have access to any data and were not involved in the analysis or interpretation of any data.

### Development of Sociotechnical Map

Data from the interviews pertaining to the data handling and flow process, including people and systems involved, were used to develop a data flow diagram of the sociotechnical system (by DK), which was initially based on an early process diagram of the telecare system provided by the principal officer at GCC HSCP. This map was then reviewed internally by the research team continuously (DK, ML, ED, and KK) before being validated for accuracy by 3 GCC stakeholders. Data were also used to develop a comprehensive description of staff roles, systems involved, the range of service user equipment, and the data used within the sociotechnical system.

### Thematic Analysis of Barriers and Facilitators

All data were analyzed inductively using Braun and Clarke’s thematic analysis procedure [12] supported by the NVivo (Lumivero) analysis software, focusing on the identification of barriers and facilitators to the data handling process. Transcripts were examined for key themes and coded with labels that were then developed further by DK, ED, NW, and ML into a codebook over 3 iterations until a final codebook was agreed. This codebook was then used for validation, with 30% of the transcripts independently coded by 1 researcher (DK) plus 2 others (NW and ED), who then met and compared coding. Agreeability was high enough that the remaining transcripts were coded by 1 researcher (DK), who used the results to develop the thematic map (Multimedia Appendix 2). Data analysis occurred between November 2022 and January 2023.

### Ethical Considerations

This study was granted ethical approval by the University of Strathclyde Ethics Committee (ID 1937; approved on July 20, 2022). Participants provided informed consent at each stage of the study after reading the relevant PIS and signing a consent form, based on the University of Strathclyde’s standard template. The PIS and consent forms informed participants that their participation was voluntary, that their data would be anonymized, and that they would remain anonymous in the reporting of the research. Participants were informed about their freedom to refuse or withdraw their participation. Participants did not receive any compensation for taking part in the study. The Consolidated Criteria for Reporting Qualitative Research (COREQ) guidance was followed for our reporting of this study (Checklist 1).

## Results

### Participants

In total, 14 people participated; 12 (85.7%) people by interview and 2 (14.3%) people by questionnaire. Most were female (n=8, 57%) and aged between 28 and 66 years. The median age was 47 (IQR 42.75‐56.25) years. Most worked in the GCC HSCP (n=11, 78.6%) and had 0‐15 years’ experience in their role, with a median of 5 (IQR 2.25-12) years’ experience (Table 1).

**Table 1.** Participant demographics (N=14).

Characteristics	Value
Age (y) median (IQR; range)^a^	47 (42.75‐56.25; 28-66)
Years’ Experience (y) median (IQR; range)	5 (2.25‐12.0; 0-15)
Gender, n (%)	
Woman	8 (57.1)
Man	6 (42.9)
Job role, n (%)	
Management	6 (42.9)
Operational	7 (50)
Not Stated	1 (7.1)

a N=2 participants did not disclose their age

### Sociotechnical Map

Figure S1 in Multimedia Appendix 3 illustrates the full complexity of the telecare services system explored in this study, as described by participants. As illustrated in Figure S2 in Multimedia Appendix 3, data are frequently being passed between various teams (4 internal teams and 5 external teams). This typically results in the alarm service team being a chokepoint for data, often relaying data between systems and teams, as not all teams will have direct access to the necessary information within all systems. As illustrated in Figure S3 in Multimedia Appendix 3, most of the data are concentrated in the Care First system, which is populated by HSCP Management and used by alarm services. In total, there are 6 primary systems that share data, but these data are not automated and have to be manually updated and maintained by the various teams. This issue is compounded further by the variety and volume of data sources that feed data into these systems, as presented in Figure S4 in Multimedia Appendix 3. This provides an overview of the complexity of the sociotechnical system where many of the barriers arise, but also the opportunities to improve and streamline the data in the future.

### Theme 1: Challenges for Predictive Data-Driven Approaches in Telecare

The main challenges reported in this theme are related to suboptimal systems and equipment, inefficient recording and use of data, barriers to inclusive telecare, and limited resources and support for exploiting the data.

#### Suboptimal Systems and Equipment

In total, 7 participants reported that there was no single standardized process or technology used in telecare, leading to significant regional variation, and there were calls for the development of a minimum dataset which could be used across Scotland (which has since been introduced). Even definitions, such as what is defined as a fall, can vary, making direct comparison between regions difficult, as what one region may record as a fall may not be recorded as such in a different region:

*I think that platform providers should be using a defined list that should be set by either the Scottish Government, or even the UK governments…if we have these standard criteria that we’re all using…we’re all comparing like for like. The problem is just now, we’re comparing apples, oranges, pears, and grapes…it’s like every fruit there.*

[Participant A10, Operational Staff]

It was reported that with new technologies and a lack of standardization, platform providers often developed technology that was only functional within single organizational ecosystems, often using stand-alone portals. This meant each technology required its own software, on which staff require specific training on how to use, often with no option to transfer the data out of the system. Participants reported this as particularly problematic, as when technology stops being supported and used, it can mean losing access to all the data contained within that portal, raising significant concerns around what happens when systems and technology are retired:

*… if you have three different suppliers, you have three different device management portals, which is a lot to manage…what does happen when they update the portal and what happens to the data that’s already on there?*

[Participant A7, Operational Staff]

There was a perception by participants that the market for telecare devices may have stagnated, with 1 participant stating that the market had not changed for several decades. Systems that were designed for care settings (where staff members were typically on site and could respond quickly), as opposed to a home environment, were still common:

*There’s some real limitations about the devices we got, the devices we got have really haven’t changed over the past 30 years and they were actually made to suit in a care homes or residential areas where staff are on site and they could respond to it quickly.*

[Participant A3, Managerial Staff]

#### Inefficient Recording and Use of Data

In total, 7 participants reported that sensor and service user data are currently underused and rarely used proactively. An overreliance on free-text entry and an inability to search or filter data make retrieval difficult when manually generating reports that get used for reporting monthly activity and service user needs. The over usage of free-text entry into records, including making notes intended to be read by other team members, resulted in misunderstanding or misinterpretation of entries. Manually generated reports were described as one of the most labor-intensive and time-consuming tasks by participants, and a lot of important user data regarding service user needs may not be incorporated into monthly reports and key performance indicators (KPIs) due to volume:

*Basically to do a desktop assessment for everybody that had door lights installed in the last 12 months… I had to go through it, every single call record individually…every time that it alerted the door sensors, I’d need to go in, record the outcome of the call and record… if they’d better management report functions from our data, if you know what I mean, so you can just get in at the top end, get the data you need.*

[Participant A5, Operational Staff]

*There’s so much data that’s being collected. And then if we can get a better handle and use this data, we can get far more efficient services, especially if we can implement things such as machine learning and AI to automatically analyse. And you’ll find patterns and predict what times we need new equipment and that sort of things.*

[Participant A1, Operational Staff]

Another concern was related to the lack of interoperability between the different systems in use. Participants reported many independent systems used throughout the telecare service; however, they are not all connected, which leads to inaccuracies and repetition of data. This was reported as particularly difficult in relation to external systems, where there are a lack of any data-sharing agreements:

*…I think that that [one system] talking to [another system]…so the person doesn’t need to give that information three or four different times…all of those services talk to each other. They would have the bigger picture on that person, which would definitely improve the care that that person receives and prevent a lot of people been admitted to hospital when they don’t need to be admitted to hospital.*

[Participant A7, Operational Staff]

Staff members not having access to data also affected its use. Participants reported that communication between teams was often superfluous, as staff often had to contact people in different departments to relay necessary information to those who do not have access to certain systems. For example, due to not all staff members having access to all relevant systems, the responder team would need to contact the alarms team to relay details regarding a service user and how to support them during an incident, such as the service users’ physical descriptions or impairments, the location of lock boxes containing keys for access, where the service user may have wandered, or when they may have to request details of certain medical requirements or impairments in order to support the service user during a medical incident. This was all done by staff members instead of being able to access that data directly themselves. This type of information can be vital for the responder team to know when dealing with a callout. Many participants had worked in multiple departments over time and referenced missing some of the features and data in systems they had access to in previous roles but could no longer access:

*…looking at [the main device portal] and we are just telling them this person has a medical history of dementia. They’re prone to falls, but if they were able to see that for themselves, I just think it would cut down on a lot of miscommunication…and just time doing these calls back and forth to pass on information because you could pass a job and then you forgot to tell them all you actually need to come back here to get a key to then go back to the job…technology for responders is really critical at the moment. I think we need to get them up to speed and allow them to have access to a lot more .*

[Participant A1, Operational Staff]

#### Barriers to Inclusive Telecare

In total, 2 participants reported issues around some telecare services not reaching certain populations, which may lead to inequalities. One participant surmised there was a lack of uptake for telecare services in one ethnic minority community in the city. However, the lack of data collected on service users’ ethnicity means it is not actually possible to accurately compare minority populations’ needs with those of the whole community to identify and address any potential inequality:

*For example, we are confident we see this, there’s not a huge uptake in the Asian [population] in the mix, the African population in the city…so that’s a challenge.*

[Participant A2, Managerial Staff]

The IT literacy of some service users was also a concern, as an over-reliance on new and varied technologies can leave many service users vulnerable if they do not have people to support them with technology. Concerns were reported regarding funding and the cost of procuring and implementing new telecare equipment. Participants worried that increased costs of bespoke technologies could create a 2-tier system, resulting in inequality for service users. Not all service users can afford to have the most up-to-date or relevant technologies as others, possibly resulting in an inequality of care and service:

*What doesn’t work well is the need for IT literacy. At times reliance on new forms of technology…For those with little money or those who are not able to negotiate these systems…it is denied to them…access to a landline is prohibitive financially for many people. Passive technology is less prevalent but essential for those with incapacity who cannot engage in current forms of Telecare in operation.*

[Participant C2, Managerial Staff]

#### Limited Resources and Support

High workload, understaffing, and recruitment problems were all raised by 4 participants as challenges:

*They are having problems with their support services and they are either understaffed or long term sickness…There’s simply too few staff within the service… some of the data that we are pulling off is evidencing that there there’s a lack of staffing or short staff-ness that is impacting on the service provision that the providers are actually providing to their service users.*

[Participant A9, Managerial Staff]

Furthermore, these participants felt that financial intervention from the government would be vital to continue the provision of telecare services. One particular concern, especially moving from analog to digital, was the lack of existing infrastructure required to support internet-connected devices, with limited support in the community to prioritize updating and replacing this infrastructure. One participant described how some more rural communities cannot always provide the reliable data needed to support internet-enabled devices. Older networks would still be relied upon, likely to be decommissioned soon, severely diminishing reliability, as companies may pull support for the equipment:

*In some of our, particularly, tenement flats in Glasgow, actually getting a signal with that SIM service or whatnot through some of that thick sandstone is an issue in itself. It’s issues that we’re grappling with in terms of how we ensure that those who want the service can get it and get it in a reliable fashion.*

[Participant A4, Managerial Staff]

*I would also say that others have struggled with it…We did look at the Highland Project and model and it took a lot of investment, but the public didn’t particularly like it. So we’ve got to change hearts and minds as well as this technology about what the role technology can play.*

[Participant A2, Managerial Staff]

Additionally, the nonemergency use of alarms by service users affected workload, where incidents would be better dealt with by a different provider, was another issue reported by participants. One participant estimated that approximately 30% of service users may frequently trigger such alarms:

*Now, that might be ‘can you phone my son, can you phone my daughter?’ That sort of thing. It might be that there’s a medical emergency. It might be that they need a responder callout. It might be that they just need a joiner or something like that.*

[Participant A10, Operational Staff]

### Theme 2: Data Potential – Opportunities to Enable More Proactive Predictive Telecare Services in the Future

Several opportunities were identified in the analysis, which could help alleviate the challenges raised (refer to Theme 1): more efficient data organization and retrieval, integrated systems that share data across the service, and predictive and proactive data analysis tools.

#### More Efficient Data Organization and Retrieval

In total, 5 participants reported that exporting data on telecare service users into a usable format can be time-consuming. Participants suggested that data could be filtered and more easily searched if it were formatted in a more standardized way to allow for more granularity, with certain key details (such as impairments, descriptions, and particulars about a service user) separated into defined categories. Data filtering would also enable search and retrieval, allowing staff to quickly configure what is displayed, giving access to the most salient information needed. This would also allow the automatic generation of reports, but to achieve this, data standardization and adherence to data governance are required:

*…say somebody had mental health issues. And they were kind of feeling a bit down and we get specialist mental health teams, is there a way that that person recording that data, if they agreed to it and then that getting filtered amongst the team…and then they could be some sort of follow up at the local level whether could be some sort of triage and things like that.*

[Participant A5, Operational Staff]

#### Integrated Systems That Share Data Across the Service

In total, 7 participants reported that various pieces of information about service users and their access to different telecare and social care services were recorded in a variety of different systems, resulting in staff searching across different systems to try to get the full picture of a service user and their needs, often manually. For example, when a service user is assessed by a member of staff, or if they fall, an ambulance is called, or if they are admitted to hospital, all of this is recorded, but not necessarily in all the relevant systems. Participants reported that it is very difficult to track what happens to a service user over time across different services as a result. Participants commented that a person’s Community Health Index (CHI) number (the unique health identifier all citizens have in Scotland) could be more consistently used across the various systems to enable data linkage between systems, which would result in better modeling of that service user’s health and service use journey:

*If you had that data and it was really good data…you would then inform at a national level with a business case, but what we have right now in Scotland is 32 different services…Having that where you could standardize a telecare service? And have all that data, all in the one place…some of that service that we all deliver in the same way. That would just be ideal.*

[Participant A7, Operational Staff]

Furthermore, by leveraging the popularity of current technological products that some service users already have access to (eg, fitness trackers and cloud-based virtual assistants), participants felt that more diverse datasets could be explored alongside those already in use and built into future predictive models:

*Somebody I think is far more likely than pre-pandemic to now have a smart speaker in their home to be able to connect with family and potential carers through that. I think that’s one of the avenues we can leverage in the future to make telecare as a concept perhaps more palatable to service users who would be I guess more traditionally resistant to have it, not imposed on them, but the fact that they have to have a box in their home which is a signal that they are declining in health, as opposed to having an already existing technology which can be purposed to provide a telecare service.*

[Participant A4, Managerial Staff]

*As a much more a commercial offering for families who don’t really need a community alarm service direct, so actively encourage them to think about the Alexa…how you can integrate technology. It’s now very well used in homes to actually do the same thing.*

[Participant A2, Managerial Staff]

#### Predictive and Proactive Data Analysis Tools

One of the most called-for changes from participants (n=7) was the ability to proactively use data for service planning. The telecare service was frequently described as reactive by participants, with a focus on *“crisis management”* [Participant A3, Managerial Staff]. Data on service users’ needs across their lifespan (including their equipment and service use and needs) were seen as vital for staff members to make decisions on their care in a more proactive and personalized way. Although these data are technically available, staff must manually trawl through multiple disconnected systems, reports, and plain text fields, which is slow and lacks ambition. By making data easier to categorize and access, many processes, such as generating reports, can be streamlined or automated. With the use of predictive models, these data can be better used to identify and predict service users’ needs, so resources can be focused where they are needed most:

*We’ve moved away from any preventative…services now to very much crisis management. What happens now, how do we react to it…And this is a major weakness…quite often we get telecare referrals where the person is actually that close to going into care…*

[Participant A3, Managerial Staff]

*[One service user] ended up actually having some falls that the telecare service wasn’t able to prevent because it was reliant on her being able to push this pendant…Currently, we don’t know somebody has fallen until they push the button. I think that there is a lot of scope in how we use data in an anticipatory fashion to enhance someone’s care and linking that to services...*

[Participant A4, Managerial Staff]

## Discussion

### Principal Findings

This study sought to explore a real-world telecare service by mapping the sociotechnical system and exploring the barriers and opportunities to make this service more data-driven and proactive. This was conducted qualitatively (interviews, questionnaires, and systems mapping) with 14 participants from the largest HSCP in Scotland (Glasgow City, GCC). A complex sociotechnical telecare system emerged from the study, comprising diverse staff roles, service users, multiple data systems, and equipment. Key challenges to implementing predictive telecare included: suboptimal systems and equipment, underused data, barriers to inclusivity affecting specific populations and those with limited IT literacy, and constrained resources and support for being data analytics active or ready. Participants also identified substantial opportunities to advance predictive telecare through a more structured and integrated approach to data management, including enhanced data organization and retrieval, improved cross-platform interoperability, and the application of predictive analytical tools tailored to service user needs.

While technology is widely and routinely used throughout the telecare service, the entire system requires a more joined-up and organized way to manage and use the diverse and distributed data within and across organizations, which can be organizationally challenging. This has also been identified with home care data in Canadian systems [13]. Automation is limited within telecare currently, with most processes relying heavily on staff performing manual activities, such as writing case notes, updating and checking files, and cross-checking data from multiple different interactions with the service users at different points in time. The current system requires significant collaboration and communication between staff working across different departments, especially when systems are not accessible to all. One study in the Netherlands exploring social care data use identified high variation between regions in the social care data, data infrastructure, and processing of data, echoing the results of this Scotland-based study [14]. There is growing recognition that the design of social care and telecare data management systems needs to improve and could be optimized with the aid of more careful consideration of Human Factors [9,15,16]. Human Factors takes a systems approach to understanding and designing health and social care, and this can help to identify where data management changes need to be made.

Data linkage is an important factor in ensuring telecare data can be used to predict adverse events and link them to health outcomes (which are not always recorded in telecare datasets). This could result in models built to flag service users’ risk factors, as well as the resource implications for different people with different levels of telecare equipment and services. A review by de Bell et al [17] identified that effective data sharing is reliant on several factors, including goal setting, interprofessional relationships, having a policy context to develop a shared vision of care, using technology as a tool for sharing data (not as an all-encompassing solution), and data users having full awareness of the various systems used. Given there is evidence to suggest that both professionals and citizens believe that their health and social care data should be shared between relevant settings for direct care purposes [18], it is recommended that HSCPs develop joint data-sharing agreements to link health and social care data. This could result in health outcomes and cost savings of telecare services being better demonstrated. However, evidence suggests that issues, such as privacy and confidentiality, appropriate use of data, sharing with relevant organizations (eg, not private companies), and unintended consequences of data-sharing (eg, raised insurance costs if other organizations find out about a health condition), are concerns of service users and patients [18], which would need to be considered in the development of data-sharing agreements.

The telecare devices of the future promise to offer more ways to actively monitor service users and their homes, as well as new ways of interacting with the systems (eg, voice and cloud-based virtual assistants) [19]. With more advanced sensing technology and interaction devices, the amount and diversity of data have the potential to increase. The ability to capture more about a person’s activity, mood, sleep, diet, and exercise, as well as environmental data (eg, room temperature and time of day) and use this data to predict how well a person is and how likely they are to experience an adverse event may occur [20]. Predicting the likelihood of those events presents the possibility of building models to intervene before they happen. Steps could then be taken to avoid these events and provide services in time to minimize possible negative outcomes (eg, being injured or hospitalized).

As we move forward into a fully digital telecare service within this Scottish setting [21], it is critical that efforts are made to future-proof the data collection and management systems to make them analytics-ready. Incomplete or unstructured data can hinder the development of statistical or machine models, which can enable more preventative and predictive models of care [22,23]. A key area for consideration is the development of templates and protocols for structuring datasets to make them robust and complete, resulting in the input and outcome variables being easily identified and used in machine modeling [24]. Relevant multistakeholder training will be required to ensure that all partnership organizations agree on a minimum dataset, allowing for national consistency yet allowing room for appropriate regional variation. Without this training, the risk of inaccurate and incomplete data coding is high, possibly leading to misinterpretation of data items and data meaning. Furthermore, lack of awareness of the importance and benefits of a more consistent approach to data recording and use to the service and care that service users receive is also a risk [25].

The next steps to progress this work would require further stakeholder engagement to translate the findings into a more concrete staged roadmap, including, for example, interoperability pilots and longer-term predictive pilots both with GCC and through reaching out further to other HSPCs nationally. This might include (1) developing standardized telecare minimum datasets and promoting their utility to encourage return of such benchmarking data; (2) encouraging HSPCs to create better linkage between different systems that store different data that when linked could reveal predictive opportunities; (3) upskill staff on data linkage, data management, and analytics capabilities; and (4) creating usable dashboards that different levels of staff can use to generate queries and interrogate telecare data. While this study focused on falls and community alarm data, there are other use cases in telecare for more predictive services that could learn from this work, such as predicting hospital readmissions and medication adherence.

### Strengths and Limitations

One of the strengths of this study is that it contributes to the distinct lack of research on the sociotechnical aspects of data prepared for future use within social care and, more specifically, telecare services. Machine learning and AI are increasingly being explored in health care settings, but data-driven social care has until now been relatively unexplored. The data collected during this study were incredibly rich, providing quality as well as a range of insights from professionals from different staff groups and with many years of experience in a field that is underrepresented in the published literature. The main limitation was that participants were sampled from only 1 geographical area in Scotland and were selected purposively by GCC based on their role, which might have led to some bias in the sample (eg, participants may have wished to present themselves or their organizations positively throughout the interviews). However, given that the interviewer had no previous relationship with the participants and did not work in this respective field, this minimizes the risk of this, and participants were explicitly informed that all comments would be anonymized and their participation would be confidential. Furthermore, the region sampled was the largest in the country. Given that participants reported huge variation in how telecare services are run and how data are collected, it would be beneficial to collect data from participants across Scotland to corroborate and add to these accounts. Finally, we did not include service users themselves as participants in this study, as the aim was to explore professionals’ perspectives. This is also a rich area for future work that should be explored.

### Conclusions

This study explored telecare professionals’ perspectives and experiences of the data collected and used in one telecare community alarm service in Scotland. The sociotechnical system map showed that the telecare service is complex. Like other research findings, there are many separate systems for storing data about service users and their use. The data are often incomplete, disconnected, and underused, especially in relation to more predictive analytics. Future research should continue to explore the challenges and opportunities relating to data analytics readiness within telecare services at a larger, more national scale and identify ways to make organizations and systems more analytics-ready in the future. They should also strive to improve linking social care datasets with health outcome datasets to more fully realize the benefits of more predictive and proactive telecare services in Scotland, the United Kingdom, and beyond.

## Supplementary material

10.2196/85056 Multimedia Appendix 1 Interview schedule.

10.2196/85056 Multimedia Appendix 2 Themes diagram.

10.2196/85056 Multimedia Appendix 3 Theme 1: the sociotechnical system map and tables.

10.2196/85056 Checklist 1 COREQ checklist.

## Abbreviations

AI: artificial intelligence
CHI: Community Health Index number
COREQ: Consolidated Criteria for Reporting Qualitative Research
GCC: Glasgow City Council
HSCP: health and social care partnership
IQR: Interquartile Range
KPI: Key Performance Indicators
PIS: participant information sheet

## References

[1] Saito N, Menga D (2015). Ecological design of smart home networks: technologies, social impact and sustainability.

[2] Milligan C, Roberts C, Mort M (2011). Telecare and older people: who cares where?. Soc Sci Med.

[3] (2023). Insights in social care: statistics for Scotland, support provided or funded by health and social care partnerships in Scotland 2021/22. Public Health Scotland.

[4] (2023). The digital shift and its impact on the telecare sector in England. UK Government.

[5] Díaz JME, Sánchez-Cervantes JL, Colombo-Mendoza LO, Alor-Hernández G, Martínez IL (2020). An architecture for an IoT-based telecare system for the elderly using big data analytics. Res Comput Sci.

[6] Barlow J, Singh D, Bayer S, Curry R (2007). A systematic review of the benefits of home telecare for frail elderly people and those with long-term conditions. J Telemed Telecare.

[7] Thorpe J, Barrett D, Goodwin N (2015). Examining perspectives on telecare: factors influencing adoption, implementation, and usage. SHTT.

[8] Dash S, Shakyawar SK, Sharma M, Kaushik S (2019). Big data in healthcare: management, analysis and future prospects. J Big Data.

[9] Guise V, Anderson J, Wiig S (2014). Patient safety risks associated with telecare: a systematic review and narrative synthesis of the literature. BMC Health Serv Res.

[10] (2023). Care in the digital age: delivery plan 2023-24, Scotland’s digital health and care strategy. Scottish Government.

[11] Anderson E, Lennon M, Kavanagh K (2024). Predictive data analytics in telecare and telehealth: systematic scoping review. Online J Public Health Inform.

[12] Braun V, Clarke V (2006). Using thematic analysis in psychology. Qual Res Psychol.

[13] Elliott J, Gordon A, Tong CE, Stolee P (2020). “We’ve got the home care data, what do we do with it?”: understanding data use in decision making and quality improvement. BMC Health Serv Res.

[14] Bos VL, Klazinga NS, Kringos DS (2024). Social care data and its fitness for integrated health and social care service governance: an exploratory qualitative analysis in the Dutch context. BMJ Open.

[15] (2018). Human factors for health and social care. Chartered Institute of Ergonomics & Human Factors.

[16] (2011). Health care comes home: the human factors. National Research Council Division of Behavioral and Social Sciences and Education.

[17] de Bell S, Zhelev Z, Bethel A, Coon JT, Anderson R (2024). Factors influencing effective data sharing between health care and social care regarding the care of older people: a qualitative evidence synthesis. Health Soc Care Deliv Res.

[18] Fylan F, Fylan B (2021). Co-creating social licence for sharing health and care data. Int J Med Inform.

[19] Chaturvedi U, Chauhan SB, Singh I (2025). The impact of artificial intelligence on remote healthcare: enhancing patient engagement, connectivity, and overcoming challenges. Intelligent Pharmacy.

[20] Lebea K, Leung WS, Nagar A, Jat DS, Mishra D, Joshi A Intelligent Sustainable Systems Worlds4 2024 Lecture Notes in Networks and Systems.

[21] (2025). Care in the digital age: delivery plan 2025 to 2026. Scottish Government.

[22] Sedlakova J, Daniore P, Horn Wintsch A (2023). Challenges and best practices for digital unstructured data enrichment in health research: a systematic narrative review. PLOS Digit Health.

[23] Tsvetanova A, Sperrin M, Peek N, Buchan I, Hyland S, Martin GP (2021). Missing data was handled inconsistently in UK prediction models: a review of method used. J Clin Epidemiol.

[24] (2025). Open data user guides: structuring a dataset. statistics.gov.scot.

[25] Housawi A, Lytras MD, Lytras MD, Housawi A, Alsaywid BS, Aljohani NR (2025). Next Generation eHealth.

