# Supplementary material for: Exploring the Barriers and Opportunities for a More Predictive Data-Driven Telecare Service: Qualitative Study in Scotland
Source: JMIR Form Res. 2026 Feb 27;10:e85056. doi: 10.2196/85056 (PMC12954676; doi:10.2196/85056)
Supplement: Multimedia Appendix 1 [file formative-v10-e85056-s001.docx]

## Data Driven Telecare Services: Qualitative Exploratory Study Interview Schedule

*Interview questions and prompts may vary depending on the type of participant being interviewed (I.e., management level versus operational)*

Intro

Thank you for agreeing to take part in this interview.

The aim of this chat is to help us understand from your POV what the current (and potential future) barriers and opportunities for the more meaningful use of data for preventative and predictive data driven telecare services. The interview will take around 30-45 mins.

**Q1. Briefly, can you remind me what your role is and roughly what kind of tasks do you mainly undertake (operational, data management, service delivery, management, call handling, responding)?**

Examples of roles fit (not exclusively) into the following Categories (adapt during interviews if appropriate)

| General | Operational | Management |
| --- | --- | --- |
| Project Team:  Service Manager, Principal Officers    Localities / Referrers:  Service Manager, Team Leader, Social Worker    External: Housing (RSL managers), National TEC Team, Benchmarking Lead | Telecare Team: Senior Officer, Resource Worker    Alarms Team: Service Manager, Senior Admin, Duty Senior, Call Handler, Responder    Finance – Principal Officer    Welfare Rights – Senior Officer    Business Admin - Manager | Assistant Chief Officer    Heads of Service |

**Q2. Can you describe your typical service user?**

| General (all roles) Prompts |
| --- |
| Age, location, conditions, risks,    How frequent do service users require callouts?    Do they have emergency contacts listed? |

Note: This project is specifically about the data regarding telecare services and how it is used and more specifically how we can better use that data for things like prevention and prediction and better service allocation.

**Q3. Can you tell me a bit about your actual experiences with telecare / telehealth services and what you think of them?**

Generally, but also specifically to your role and/or at Glasgow City HCP

| General (all roles) Prompts |
| --- |
| What is your professional experience with telecare / telehealth services (what works, what doesn’t)?    What telecare services do you manage/use?    Have you had any experience with the following telecare/telehealth services in your role? (CareFirst, Home Care, Clinical Portal, PNC, eclipse? Device management portal?)    How effective do you think these services are currently? (Managing risk, getting the right assistance?)  Positives and improvements  negatives and barriers    Prompts: safety, health benefits, impact on confidence, cost, visual display of illness/incapacity, technology issues, data security, IT literacy |

**Q4. What do you know specifically about the data which is generated from telecare / telehealth services in Glasgow City HCP?**

Are you familiar with:

| General | Operational | Management |
| --- | --- | --- |
| Data systems- types of databases or systems where data is used    Data flow – who sees what and when? (Both inside and outside the HCP)    What data is gathered and used? (Is there any personalization or predictive data driven telecare) | What Information is held about people, their condition and events that happen (e.g., falls, wandering)? | What Information is shared with technology providers, social care providers and health care providers? |

**Q5. What data do you use on a day-to-day / weekly basis in relation to telecare services? And what do you use it for?**

Think about data you use for things such as:

| General (all roles) Prompts |
| --- |
| Data used when liaising with other services?  Patient Details?  Care planning?  Auditing?  Budgeting? |

**Q6. Is there any data that is currently missing, or you think should be collected?**

| General (all roles) Prompts |
| --- |
| In particular as we move to digital from analogue |

**Q7. How do you think the data generated from telecare / telehealth services be better used in the future?**

| General (all roles) Prompts |
| --- |
| How you think data could be used to identify needs better?  (Can we be more proactive or predictive?)    What do you think of the feasibility of Linking with other data outside social care?    Any changes you would like to see made?    Prompts: real time monitoring, individual care, safety monitoring, predictive modelling, service planning, confidentiality, consent, data quality etc. |

**Q8. Is there anything else you would like to mention that we should take into consideration?**

 Outro

Thank you for taking part, this is useful to help us. Your responses will be treated as anonymous as outlined in the information and consent sheets provided.

Please get in touch by EMAIL if you have any thoughts after this call.

EMAIL REMINDER – [David.Kernaghan@strath.ac.uk](mailto:David.Kernaghan@strath.ac.uk)
