# Supplementary material for: Exploring the Barriers and Opportunities for a More Predictive Data-Driven Telecare Service: Qualitative Study in Scotland
Source: JMIR Form Res. 2026 Feb 27;10:e85056. doi: 10.2196/85056 (PMC12954676; doi:10.2196/85056)
Supplement: Multimedia Appendix 2 [file formative-v10-e85056-s002.docx]

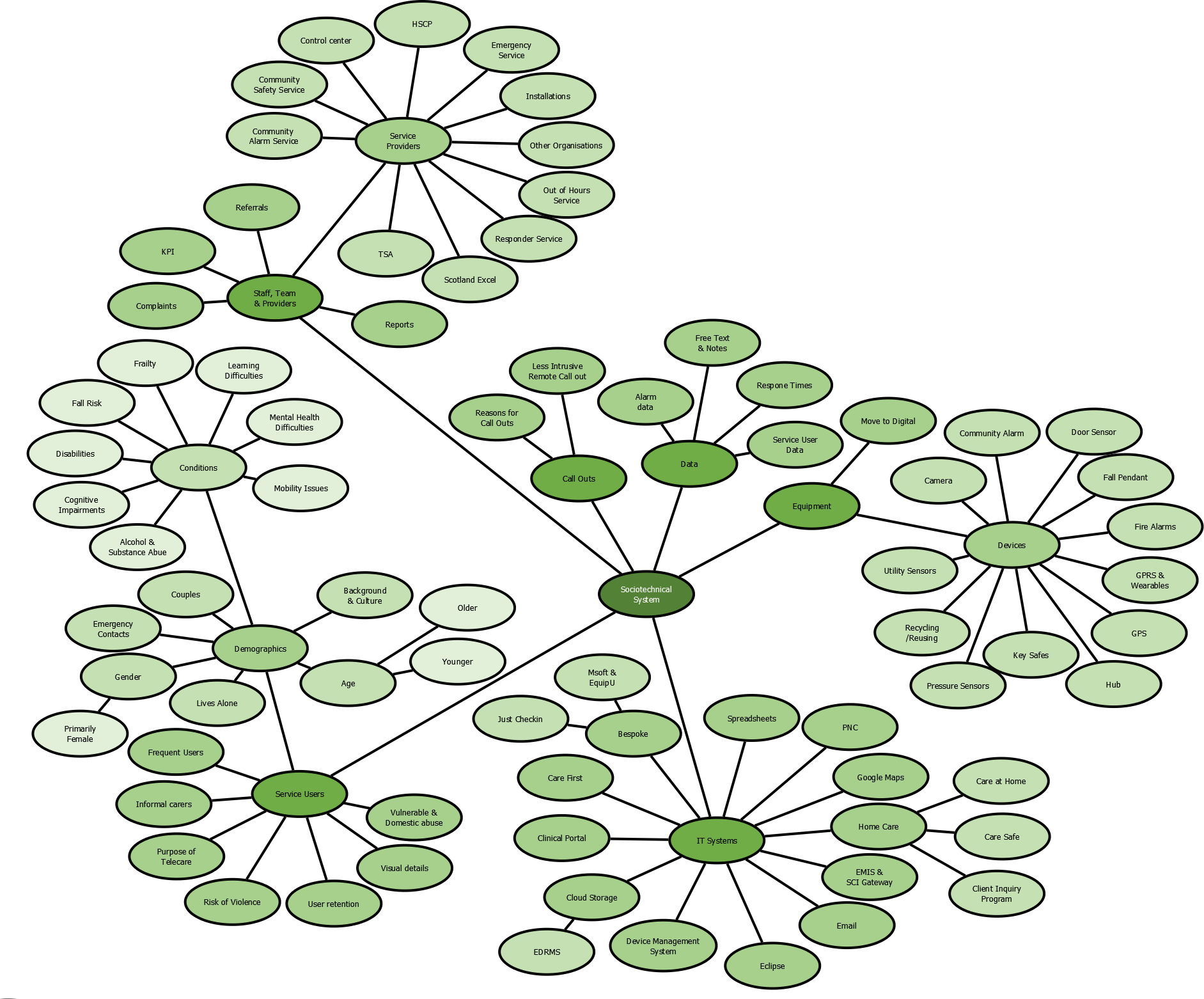


**Figure S1. Sociotechnical system thematic map.**


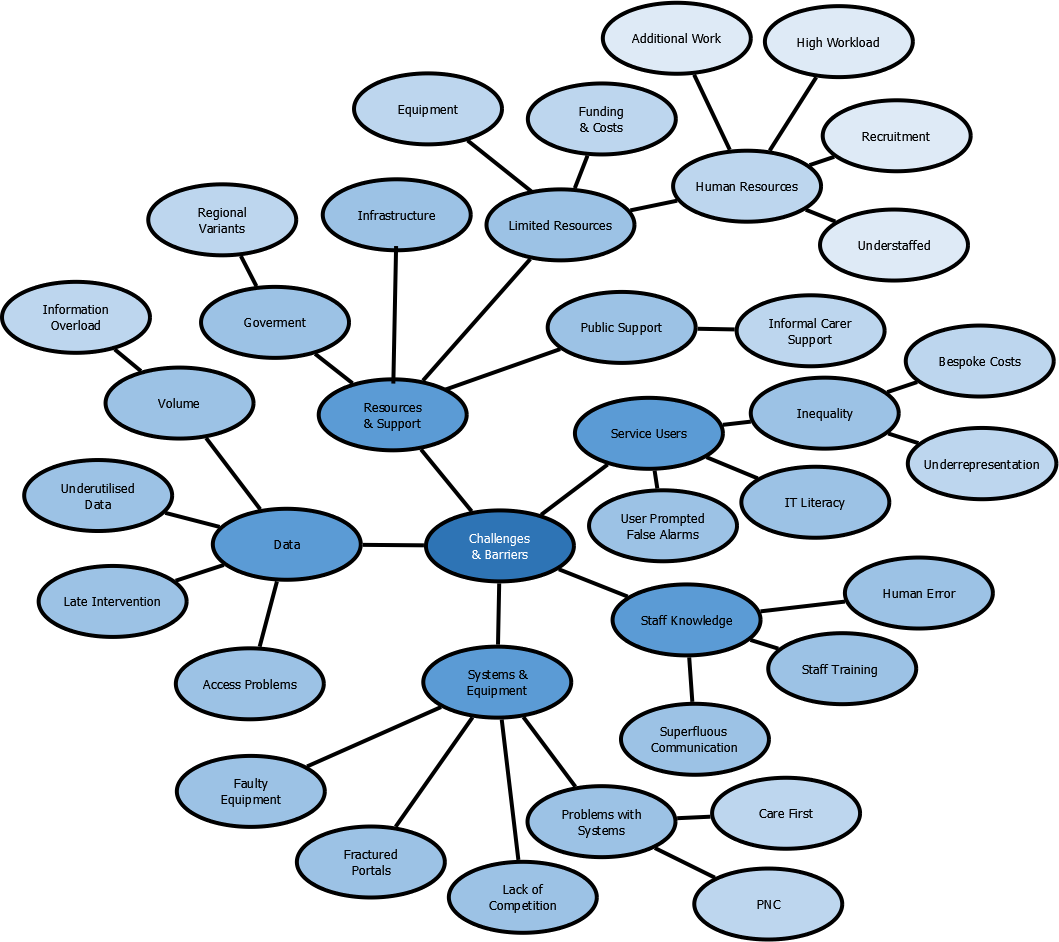


**Figure S2. Challenges and barriers thematic map.**


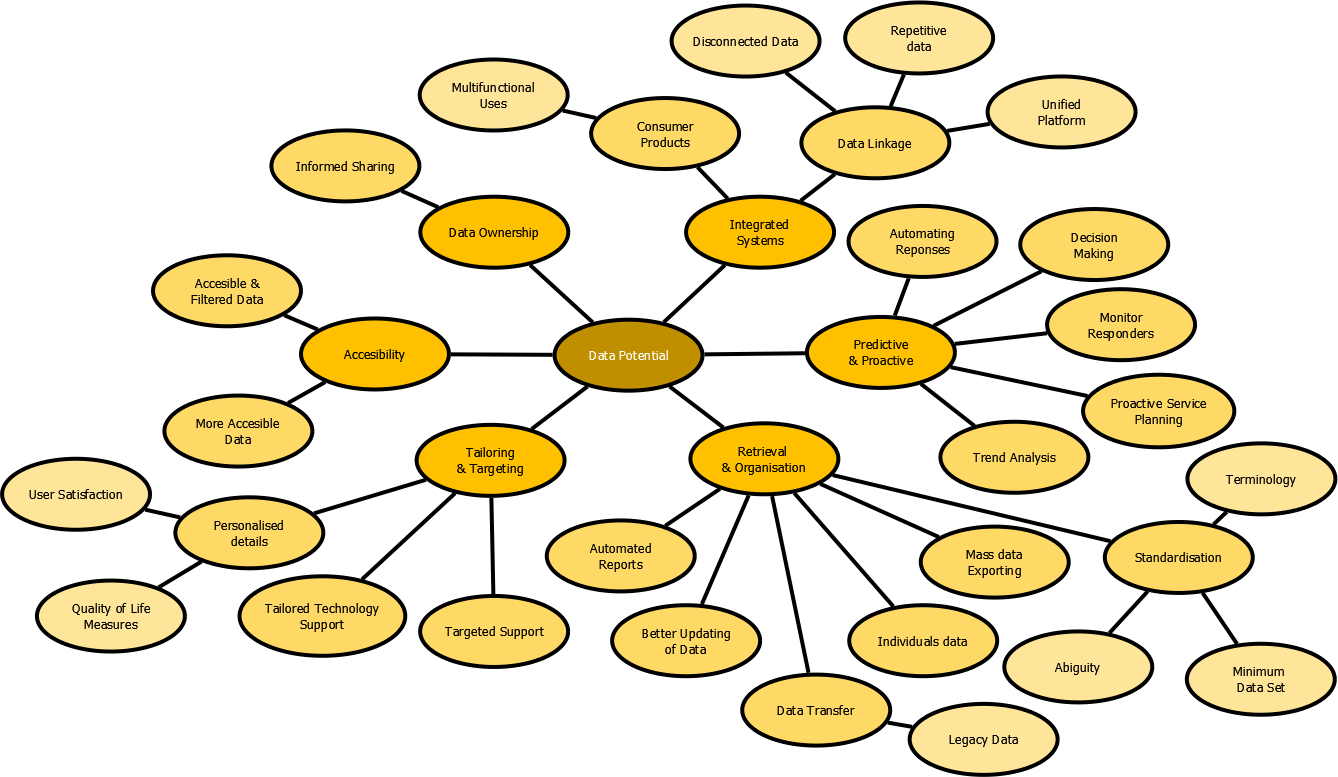


**Figure S3. Data potential thematic map.**
