# Supplementary material for: Exploring the Barriers and Opportunities for a More Predictive Data-Driven Telecare Service: Qualitative Study in Scotland
Source: JMIR Form Res. 2026 Feb 27;10:e85056. doi: 10.2196/85056 (PMC12954676; doi:10.2196/85056)
Supplement: Multimedia Appendix 3 [file formative-v10-e85056-s003.docx]

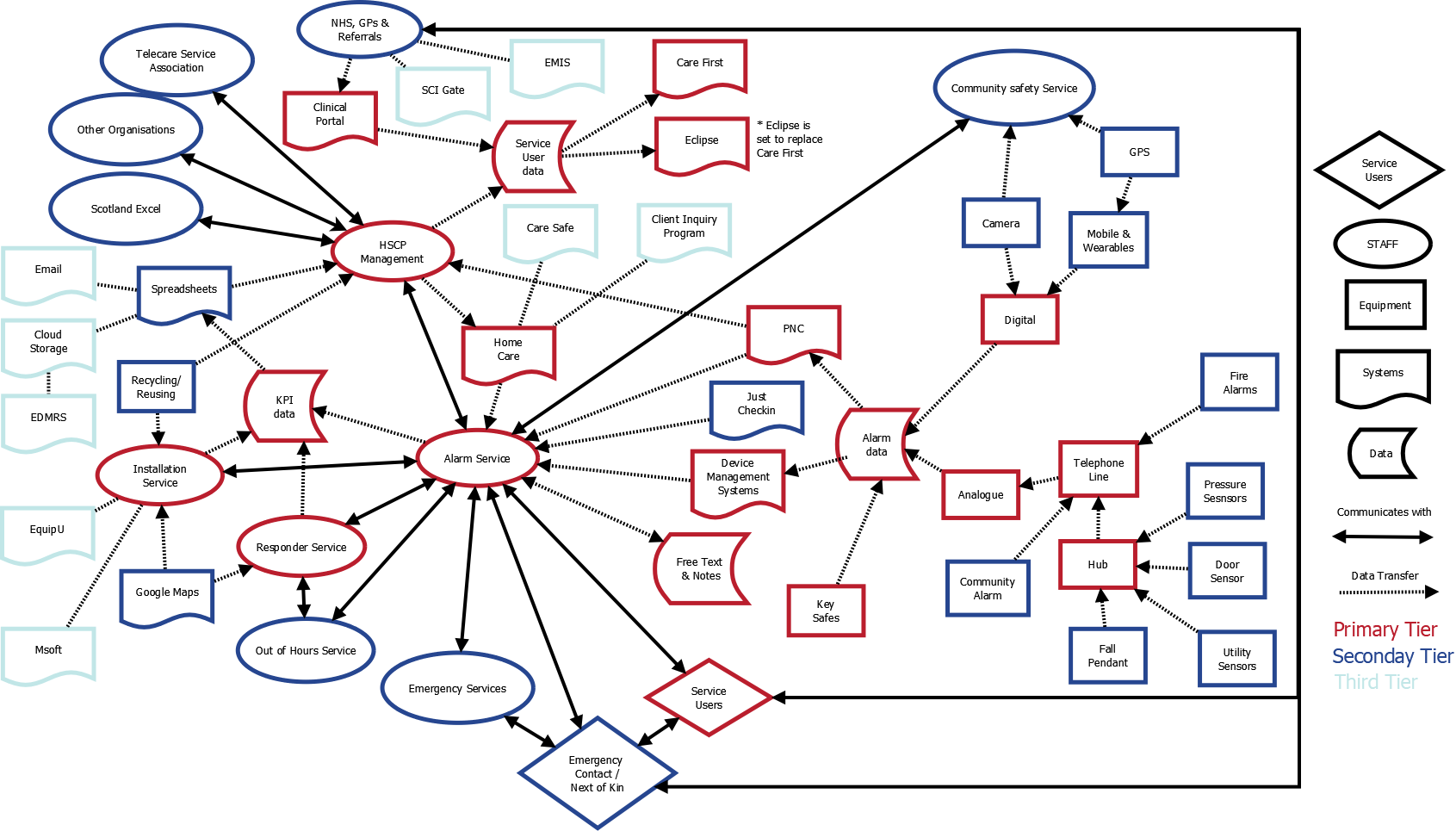


*Primary tier – directly part of the HSCP service, either staff employed by them, a primary system or equipment they use and manage*

*Secondary tier - Third party services/staff that HSCP has direct access to but is employed/managed by an outside organisation*

*Third tier - system that are entirely outside the HSCP*

**Figure S1.** Tunstall Sociotechnical system full data flow diagram.

**
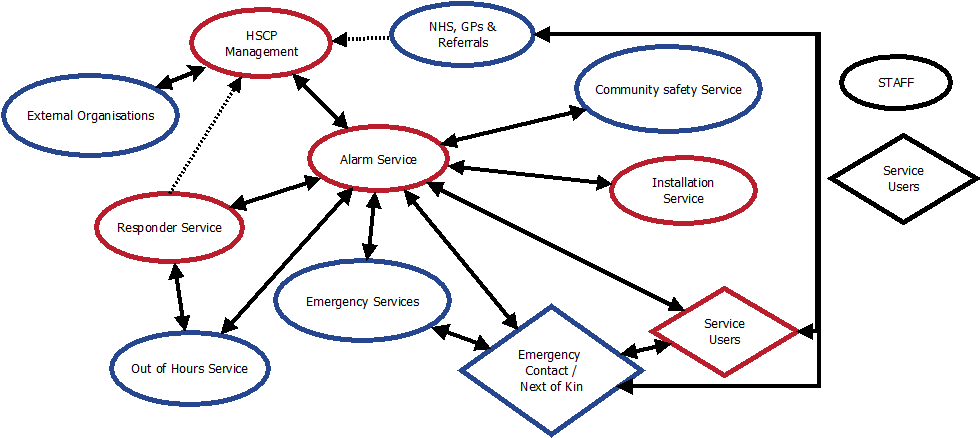
**

**Figure S2.** Tunstall sociotechnical system staff diagram.


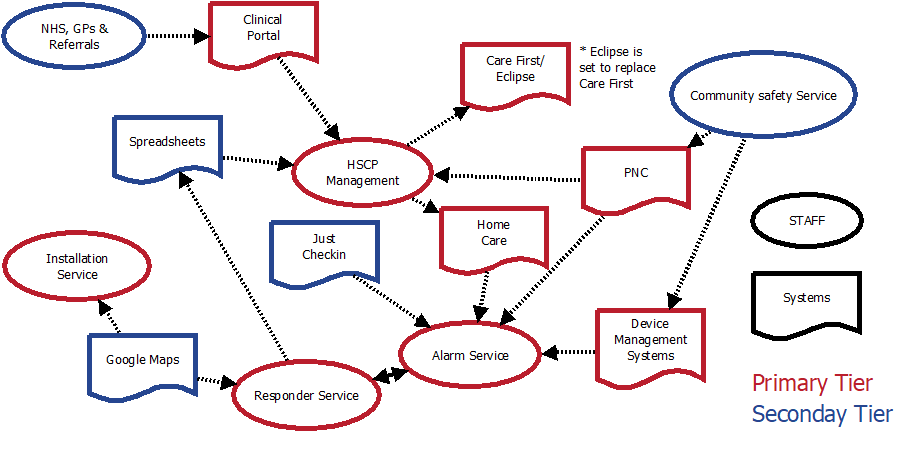


**Figure S3.** Tunstall sociotechnical system systems diagram.


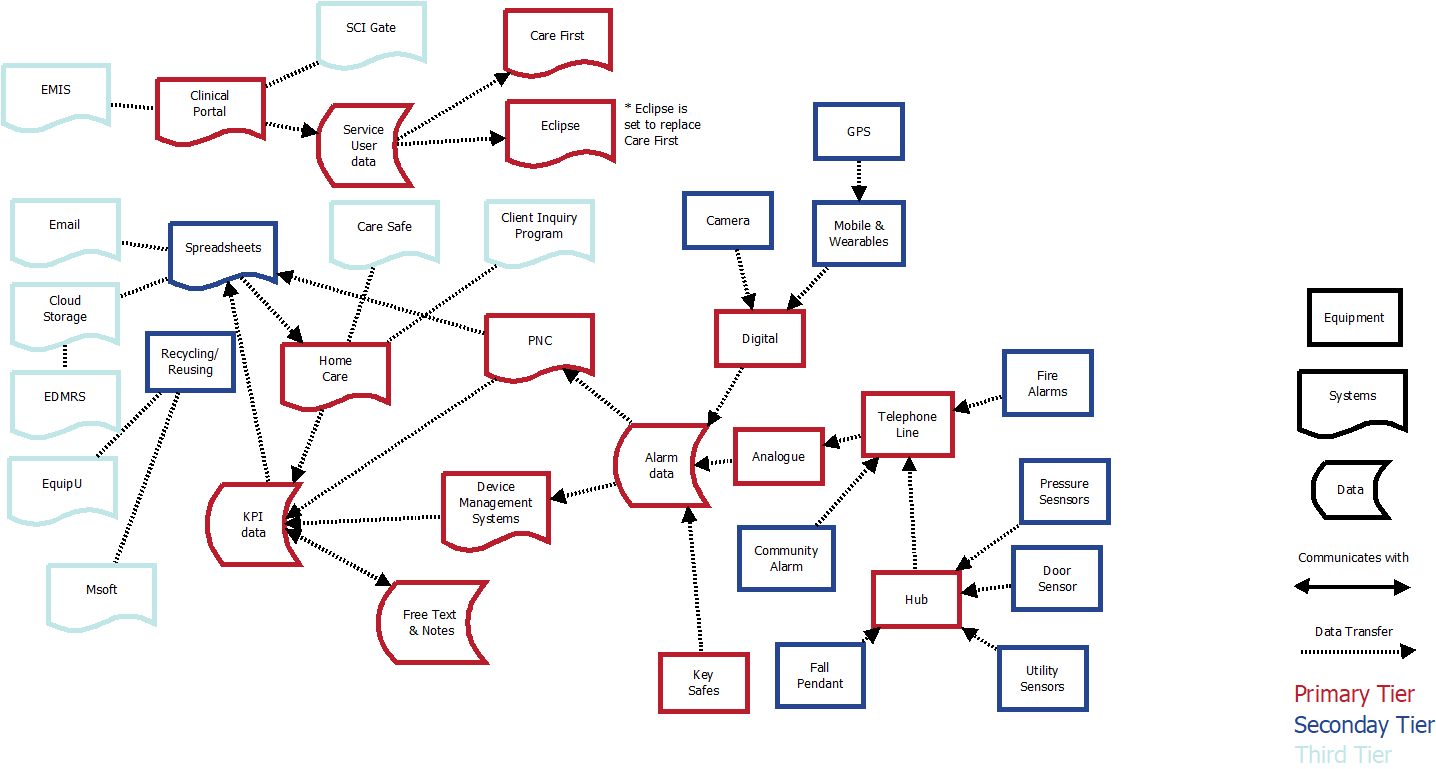


**Figure S4.** Tunstall sociotechnical system data diagram.

**Table S1. Description of staff roles.**

| **Primary Roles** | **Description** |
| --- | --- |
| Community Alarm Service (Control Center) | The main central service that deals with triggered alarms and calls |
| Responder Service (aka Call Out Service) | Service that sends staff out to check on and support service users at their home |
| Telecare Installations | Installation Team and anything related to installation of telecare innovation |
| Out of Hours Service | Subdivision of Responder Service. Service that responds to calls outside normal business hours. |
| HSCP | Glasgow city health and social care partnership |
| **Secondary roles** | **Description** |
| NHS & GPs | Primary Healthcare Providers that directly treat Service Users. |
| Community Safety Service | Manage the GPS and CCTV systems throughout Glasgow |
| Emergency Services | Ambulance, Police and Fire Brigade |
| Scotland Excel | Scotland Excel is a governing administration organisation that offers consultation, procurement services and development programs. |
| TSA (Telecare Services Association) | Telecare Services Association. An independent organisation that defines standards for telecare services in the UK. |
| Other organisations | Other organisations mentioned that are outside the usual involved service providers. These include Soul Connect (small organization who are working alongside some of the supported living accommodation providers) or Blackwood Homes & Care () |

**Table S2. Description of primary, secondary and ad hoc systems.**

| **Core** | **Description** |
| --- | --- |
| PNC | Tunstall Alarm Receiving Centre solution PNC – Piper Network Controller. The main call and alarm monitoring platform |
| Care First | The main system for storing and managing patient data. Primary managed by the social work team |
| Home Care | Manages all phone care services including recording system, care plan information and Scheduling and monitoring. Also known as Care at Home |
| Care Safe | Sub system of Home Care that manages visits |
| Clinical Portal | The main portal for accessing patients’ medical data from the NHS. Logged by hospitals. |
| Device Management Systems | Online portals used to manage the data collected from various digital equipment. Each equipment will often have its own portal. |
| Eclipse | A new system designed as a replacement for Care First to store and manage patient data |
| **Ad Hoc** | **Description** |
| Spreadsheets | Spreadsheets such as Microsoft Excel. Primary method of managing and storing reports |
| Email | Files and data shared over email, common method for delivering reports |
| Cloud Storage | System where files are stored in cloud storage/central online servers that are remotely accessible by staff |
| **Additional** | **Description** |
| Client Inquiry Program | Only Home Care staff have access to this system but can be contacted via call to speak to them |
| Google Maps | Google Maps used by staff to find locations and addresses for the responder team. |
| EMIS | Electronic Medical Information System (health-related system used by GPs and Community health teams) |
| SCI Gate | System Connection Interface (referral management system). Healthcare exclusive systems that are not managed by Social Care team. |
| Just Checkin | Activity monitoring services that help people with dementia, learning disabilities and autism live independently |
| MSoft | A cloud-based healthcare software used in conjunction with EquipU equipment |
| EquipU | A service responsible for providing and installing equipment supplied by health and social work services to people living at home |

**Table S3. Description of service user equipment.**

| **Equipment** | **Description** |
| --- | --- |
| Camera | Surveillance camera for monitoring users (such as CCTV) |
| Community Alarm | General alarm that can be triggered by user to contact the alarm centre. Can be automated, most often requires users to trigger using a button. |
| Door Sensor | Sensors that note when a door had been opened/left open. sometimes called perimeter sensors or exit sensors |
| Fall Pendant | Sensor that can trigger an alarm when patient has fallen. Can be automated but most often needs to be manually triggered using a button. |
| Fire Alarms | Passive Sensors that trigger in case of fire, often will alert the fire service as well |
| Mobile & Wearables | Mobile data technology (referred to as GPRSS) that allows mobile devices to connect to the internet |
| GPS | Global Positioning system, used to track a service user or responder teams’ location. |
| Hub | Central Hub located in the home that other devices connect to |
| Key Safes | Small storage boxes that hold keys to properties that are accessible to staff. Often located on the outside of the home with a number combination to open. |
| Pressure Sensors | Sensors that track when a user gets up from a lying / seated position. Bed sensors are most common but sometimes referred to as chair sensors, pad sensors, or mat sensors. |
| Recycling (reusing devices) | Regarding cycles that are recycled, reused and repurposed |
| Utility Sensors | Sensors that monitor common utility services such as electricity use, gas use and water use. |

**Table S4. Description of data used within the sociotechnical system.**

| **Data** | **Description** |
| --- | --- |
| Service User Data | Details relating to the collection of user data characteristics (age, gender, location, diagnosis, contact details, doctor details) |
| Alarm Data | Data regarding triggering of alarms (including phone alarms, fall alarms, fire alarms, door alarms) |
| Free Text & Notes | Data collected in the form of free text and notes in any of the systems |
| KPI Data | Key Performance Indicator data used for service planning and resource management. |
