# Supplementary material for: Exploring the Barriers and Opportunities for a More Predictive Data-Driven Telecare Service: Qualitative Study in Scotland
Source: JMIR Form Res. 2026 Feb 27;10:e85056. doi: 10.2196/85056 (PMC12954676; doi:10.2196/85056)
Supplement: Checklist 1 [file formative-v10-e85056-s004.docx]

| **Domain 1: Research team and reflexivity** | |
| --- | --- |
| *Personal Characteristics* | |
| 1. Interviewer/facilitator | |
| Which author/s conducted the interview or focus group? | DK conducted the interviews. |
| 2. Credentials | |
| What were the researcher’s credentials? E.g. PhD, MD | At the time of the study, DK was a PhD candidate. He has since graduated with his PhD. |
| 3. Occupation | |
| What was their occupation at the time of the study? | DK was a full-time PhD student. |
| 4. Gender | |
| Was the researcher male or female? | Male. |
| 5. Experience and training | |
| What experience or training did the researcher have? | DK had previous experience of collected qualitative and quantitative data, both during his PhD experience and outside of this on research projects. |
| *Relationship with participants* | |
| 6. Relationship established | |
| Was a relationship established prior to study commencement? | The participants and the researcher first engaged when researchers expressed interest in participating. The participants were signposted to us via a project partner in the health and social care partnership who was a collaborator on the project. |
| 7. Participant knowledge of the interviewer | |
| What did the participants know about the researcher? e.g. personal goals, reasons for doing the research | The PIS and an email intro stating that a researcher from Strathclyde was looking for people to take part in interviews on their experiences of working with telecare data. |
| 8. Interviewer characteristics | |
| What characteristics were reported about the interviewer/facilitator? e.g. Bias, assumptions, reasons and interests in the research topic | The PIS |
| **Domain 2: study design** | |
| *Theoretical framework* | |
| 9. Methodological orientation and Theory | |
| What methodological orientation was stated to underpin the study? e.g. grounded theory, discourse analysis, ethnography, phenomenology, content analysis | A sociotechnical framework approach was applied to the data and analysis. |
| *Participant selection* | |
| 10. Sampling | |
| How were participants selected? e.g. purposive, convenience, consecutive, snowball | Possible participants (n=17) were identified by a member of the project team from the GGC HSPC. |
| 11. Method of approach | |
| How were participants approached? e.g. face-to-face, telephone, mail, email | Individuals were contacted by a project team member to invite them to participate. Interested participants were contacted by a researcher via email to arrange an interview. Participants were provided a link to the PIS, consent and demographics forms to complete prior to the interview. Participants were informed that participation was voluntary, would be confidential, only the research team had access to data, and responses would be pseudo-anonymised. |
| 12. Sample size | |
| How many participants were in the study? | 14 people took part. |
| 13. Non-participation | |
| How many people refused to participate or dropped out? Reasons? | N=3 people from the original list did not participate. Reasons provided were: / No reasons were provided/No response was given. |
| *Setting* | |
| 14. Setting of data collection | |
| Where was the data collected? e.g. home, clinic, workplace | Interviews were conducted remotely using MS Teams©, Zoom© or telephone. Some participants provided response via online questionnaire. |
| 15. Presence of non-participants | |
| Was anyone else present besides the participants and researchers? | No. |
| 16. Description of sample | |
| What are the important characteristics of the sample? e.g. demographic data, date | Participant age, years’ experience, gender, working location and job role category were collected. |
| *Data collection* | |
| 17. Interview guide | |
| Were questions, prompts, guides provided by the authors? Was it pilot tested? | Participants were provided a copy of questions and prompts prior to the interview. These were not pilot tested. |
| 18. Repeat interviews | |
| Were repeat interviews carried out? If yes, how many? | No. |
| 19. Audio/visual recording | |
| Did the research use audio or visual recording to collect the data? | Yes. Interviews were recorded via the preferred platform and with a Dictaphone as backup. Participants who could not attend an interview could respond via online questionnaire using Qualtrics©. |
| 20. Field notes | |
| Were field notes made during and/or after the interview or focus group? | Field notes were taken during the interview process to quickly reference transcripts but were not included in the final analysis |
| 21. Duration | |
| What was the duration of the interviews or focus group? | Interviews lasted between 17-47 minutes each. |
| 22. Data saturation | |
| Was data saturation discussed? | No. Given the maximum potential sample provided to the research team by the project team was n-=17, and n=14 participated, data saturation was not discussed. |
| 23. Transcripts returned | |
| Were transcripts returned to participants for comment and/or correction? | No. |
| **Domain 3: analysis and findings** | |
| *Data analysis* |  |
| 24. Number of data coders | |
| How many data coders coded the data? | Three researchers were involved in data coding: one in the main analysis and two for validation. |
| 25. Description of the coding tree | |
| Did authors provide a description of the coding tree? | No. The main themes and sub-themes are provided. |
| 26. Derivation of themes | |
| Were themes identified in advance or derived from the data? | Themes were derived from the data. |
| 27. Software | |
| What software, if applicable, was used to manage the data? | NVivo© was used to manage the data, as well as OneDrive© for secure storage. |
| 28. Participant checking | |
| Did participants provide feedback on the findings? | No. |
| *Reporting* | |
| 29. Quotations presented | |
| Were participant quotations presented to illustrate the themes / findings? Was each quotation identified? e.g. participant number | Yes, in the body of the text. Participant number and job “category” are also presented per quotation. |
| 30. Data and findings consistent | |
| Was there consistency between the data presented and the findings? | Yes. |
| 31. Clarity of major themes | |
| Were major themes clearly presented in the findings? | Yes. |
| 32. Clarity of minor themes | |
| Is there a description of diverse cases or discussion of minor themes? | Yes- sub-themes are presented. |
